# Opposite action of R2R3-MYBs from different subgroups on key genes of the shikimate and monolignol pathways in spruce

**DOI:** 10.1093/jxb/ert398

**Published:** 2013-12-14

**Authors:** Claude Bomal, Isabelle Duval, Isabelle Giguère, Élise Fortin, Sébastien Caron, Don Stewart, Brian Boyle, Armand Séguin, John J. MacKay

**Affiliations:** ^1^Center for Forest Research, Université Laval, Québec, QC G1V A06, Canada; ^2^Institute of Integrative and Systems Biology, Université Laval, Québec, QC G1V A06, Canada; ^3^Natural Resources Canada, Canadian Forest Service, Laurentian Forestry Centre, Québec, QC G1V 4C7, Canada; ^4^Department of Plant Sciences, University of Oxford, Oxford OX1 3RB, UK

**Keywords:** Conifers, phenylpropanoid pathway, protein–DNA binding, R2R3-MYB evolution, transcriptional network.

## Abstract

Redundancy and competition between R2R3-MYB activators and repressors on common target genes has been proposed as a fine-tuning mechanism for the regulation of plant secondary metabolism. This hypothesis was tested in white spruce [*Picea glauca* (Moench) Voss] by investigating the effects of R2R3-MYBs from different subgroups on common targets from distinct metabolic pathways. Comparative analysis of transcript profiling data in spruces overexpressing R2R3-MYBs from loblolly pine (*Pinus taeda* L.), PtMYB1, PtMYB8, and PtMYB14, defined a set of common genes that display opposite regulation effects. The relationship between the closest MYB homologues and 33 putative target genes was explored by quantitative PCR expression profiling in wild-type *P. glauca* plants during the diurnal cycle. Significant Spearman’s correlation estimates were consistent with the proposed opposite effect of different R2R3-MYBs on several putative target genes in a time-related and tissue-preferential manner. Expression of sequences coding for 4CL, DHS2, COMT1, SHM4, and a lipase thio/esterase positively correlated with that of PgMYB1 and PgMYB8, but negatively with that of PgMYB14 and PgMYB15. Complementary electrophoretic mobility shift assay (EMSA) and transactivation assay provided experimental evidence that these different R2R3-MYBs are able to bind similar AC *cis*-elements in the promoter region of *Pg4CL* and *PgDHS2* genes but have opposite effects on their expression. Competitive binding EMSA experiments showed that PgMYB8 competes more strongly than PgMYB15 for the AC-I MYB binding site in the *Pg4CL* promoter. Together, the results bring a new perspective to the action of R2R3-MYB proteins in the regulation of distinct but interconnecting metabolism pathways.

## Introduction

During their development, and in response to biotic and abiotic stresses, plants produce thousands of metabolites, which play a wide range of roles in environmental adaptation. The synthesis and accumulation of such products result from the activity of a plethora of enzymes under the control of complex transcriptional networks. For example, the fine regulation of phenylpropanoid biosynthesis is achieved by a combination of transcription factors (TFs) from diverse families such as WRKY, bZIP, MADS-box, bHLH, WD40, and R2R3-MYBs (Zhao and Dixon, 2011). Recent studies of transcriptional regulation of specific aspects of primary and secondary metabolism have begun to define transcriptional networks underlying metabolic responses.

The R2R3-MYBs represent one of the largest families of plant TFs (Kranz *et al.*, 1998; Stracke *et al.*, 2001) and are important regulators of secondary metabolism (Dubos *et al.*, 2010, Feller *et al.*, 2011). Their DNA-binding domain (DBD) is highly conserved among angiosperm and gymnosperm plants (Bedon *et al.*, 2007) but their C-terminus, which typically contains activation and repression domains, is highly variable (Dubos *et al.*, 2010). The R2R3-MYBs have been divided into 22 different subgroups (SGs) based on their DBD and C-termini sequence features, although some of them remain unclassified (Kranz *et al.*, 1998; Stracke *et al.*, 2001). Recent evidence from model plant systems indicates that different MYBs within a subgroup may be functionally redundant. For example, several *Arabidopsis* SG7 MYBs were shown to regulate the production of flavonol glycosides by acting on similar target genes (Stracke *et al.*, 2007), and the maize ZmMYB31 and ZmMYB42 both regulate *CINNAMATE 4-HYDROXYLASE* (*C4H*) (Fornalé *et al.*, 2010). Functional characterizations have also shown that MYBs from different subgroups may act upon overlapping sets of target genes in pathways of flavonoid, monolignol, xylan, or cellulose metabolism (reviewed in Dubos *et al.*, 2010; Zhong *et al.*, 2010).

Many R2R3-MYBs have been described as transcriptional activators of genes from phenylpropanoid, flavonoid, and glucosinolate biosynthetic pathways in plants (Dubos *et al.*, 2010; Zhong *et al.*, 2010; Zhao and Dixon, 2011). Alternatively, a few of them, including members of the SG4 subgroup, have been identified as negative regulators of monolignol and flavonoid biosynthetic pathways (Tamagnone *et al.*, 1998; Jin *et al.*, 2000; Preston *et al.*, 2004; Legay *et al.*, 2007; Fornalé *et al.*, 2010). In *Eucalyptus grandiis*, two R2R3- MYBs, namely EgMYB1 (Legay *et al.*, 2010) and EgMYB2 (Goicoechea *et al.*, 2005), were shown to act as a repressor and activator of lignin biosynthesis, respectively. The putative interplay of activators and repressors including MYBs acting on common target genes has been proposed as the basis for fine transcriptional regulation of phenylpropanoid metabolism (Vom Endt *et al.*, 2002) and lignin biosynthesis (Zhao and Dixon, 2011). Activation and repression have also been attributed to the action of a single MYB gene (Bhargava *et al.*, 2010). For example, *Arabidopsis* MYB75, also known as PRODUCTION OF ANTHOCYANIN PIGMENT1 (PAP1), was shown to regulate anthocyanin biosynthesis positively in seedlings (Borevitz *et al.*, 2000) and, more recently, to regulate negatively the expression of monolignol biosynthesis genes and the accumulation of lignin in the inflorescence stems of older plants (Bhargava *et al.*, 2010). Together, these results also suggest that a dual effect may exist when considering different tissues.

Our understanding of functional redundancy and antagonistic effects among R2R3-MYBs has essentially been derived from the studies of flowering plants (i.e. angiosperms) and remains to be extended to other seed-bearing plants. The conifer MYBs *Pinus taeda* PtMYB1 and PtMYB8 were previously identified as positive regulators of monolignol biosynthesis (Bomal *et al.*, 2008). It was also shown that PtMYB14 and PtMYB15 ectopic overexpression could up-regulate isoprenoid metabolism (Bedon *et al.*, 2010), but these latter two MYBs were suspected to be potential repressors since they harbour the ERF-associated amphilic repression (EAR) motif (Ohta *et al.*, 2001). Here, evidence is presented for antagonistic action among these four MYBs on a set of common target genes in shikimate and monolignol biosynthetic pathways in *Picea glauca*. Comparative transcript profiling in PtMYB overexpression lines, diurnal variation of gene expression in secondary vascular tissues, transactivation assays, and DNA binding assays were used to identify the putative targets of *P. glauca* (Pg) R2R3-MYBs. As such, the findings show that several R2R3-MYBs in *P. glauca* may act upon common targets through activation and repression of expression in two distinct but interconnecting pathways.

## Materials and methods

### Comparative analysis and functional annotation of microarray data sets

Microarray transcript profiling data were from wild-type and transgenic white spruce [*P. glauca* (Moench) Voss] overexpressing *Pinus taeda* PtMYB1, PtMYB8 (Bomal *et al.*, 2008), and PtMYB14 (Bedon *et al.*, 2010). *Picea glauca* is an established transgenic expression system that is the most homologous available for analysing genes in the Pinaceae. The data sets are available in the public database Array Express (EBI): E-MEXP-3626 for PtMYB1 and PtMYB8 and E-MEXP-3575 for PtMYB14. These data sets were compared by using a fold change of ≥|1.4| [*P*-value <0.01, false discovery rate (FDR) of 1%] to identify shared sets of up- and down-regulated genes (Supplementary Table S1 available at *JXB* online). Annotations of cDNA sequences on the microarray were based on Rigault *et al.* (2011). Assignment to metabolic pathways from the Kyoto Encyclopedia of Genes and Genomes (KEGG) was based on annotations of TAIR homologuess (Supplementary Table S2).

### Plant material and diurnal cycle experiment

Wild-type 3-year-old spruce plantlets [*P. glauca* (Moench) Voss] were transferred to 2 litre pots, and grown for 45 d (mid-April to June) in a greenhouse under a natural photoperiod with weekly irrigation and fertilization [20/20/20 N/P/K (g l^–1^)]. Two weeks before the diurnal experiment, plants were transferred to a growth chamber (Conviron model PGW36) set to the natural summer solstice photoperiod (16h/8h, day/night) with a photosynthetic photon flux density of 860 μmol photons m^–2^ s^–1^ (constant temperature of 23 °C and 70% relative humidity).

For the diurnal cycle experiment, whole secondary xylem (2X) and bark/phloem (2P) tissue samples were collected from the main stem at 3h intervals over a 24h period. A 10cm long stem piece was cut under the first whorl from the top. A scalpel incision was made along the stem, and then 2P tissues were manually peeled off the stem and separated from 2X tissues. Both 2P and 2X tissues were rapidly cut into short pieces, immediately frozen in liquid nitrogen, and stored at –80 °C until further use. Five individual trees were sampled at each time point over the 24h period, and the experiment was performed twice.

### RNA extraction, cDNA preparation, and quantitative PCR

Total RNA was isolated as described (Pavy *et al.*, 2008). RNA concentrations were determined using NanoDrop 1000 (Thermo Scientific, Wilmington, DE, USA); RNA integrity used a 2100 Bioanalyzer (Agilent Technologies, Santa Clara, CA, USA). cDNA preparation and quantitative PCR followed Boyle *et al.* (2009) with some modifications. Briefly, 1 μg of total RNA was reverse transcribed, and a green fluorescent protein (GFP) spike-in (HM151400.1) was added as an internal control. The PCR mixture was a QuantiFast^®^ SYBR^®^ Green PCR kit (QIAGEN, Germantown, MD, USA) or a LightCycler^®^ 480 SYBR Green I Master (Roche, Basel, Switzerland). Gene-specific primers were designed by using Primer 3 software (Supplementary Table S3A at *JXB* online).

The transcript level (number of molecules) was calculated using the LRE method (Rutledge and Stewart, 2008) adapted by Boyle *et al.* (2009). Transcript levels were normalized to the geometric mean of six reference genes: *elongation factor 1a* (BT102965), *cell division cycle 2* (BT106071), *ribosomal protein L3A* (BT115036), *eukaryotic initiation factor 4E* (BT112014), *ubiquitin-conjugating enzyme* (BT109864), and *core histone H3* (BT116867). Reference gene variation was analysed with geNorm 3.5 version (Vandesompele *et al.*, 2002).

### Statistical analysis of expression data

Spearman’s correlation rank test was used to evaluate statistical dependence between candidate gene and *PgMYB* expression data. Critical ρ values in a two-tailed test at 0.05 and 0.01 significance were 0.247 and 0.325 for 2X tissue (*n*=64) and 0.223 and 0.291 for 2P tissue (*n*=78), respectively (Steele *et al.*, 1980). Due to multiple comparison tests, a *q*-value was calculated to estimate a minimum FDR (5%) to adjust the significance (*P*-value) of ρ estimates. Statistical analyses used the R package (Ihaka and Gentleman, 1996).

### Isolation of genomic sequences and AC element identification

The sequences upstream of the *Pg4CL* (BT106671) and *PgDHS2* (BT116706) genes were identified using the Universal GenomeWalker™ kit (Clontech, Mountain View, CA, USA) as described by Bedon *et al.* (2009). For *Pg4CL*, the complete coding sequence was first obtained by using 5′ rapid amplification of cDNA ends (RACE; SMART RACE cDNA Amplification Kit, Invitrogen, Carlsbad, CA, USA) in order to obtain a full-length cDNA (TA Cloning Kit, Invitrogen). Two overlapping genomic DNA fragments were then obtained with proximal primers 4CL-GSP1 and 4CL-GSP2, and distal primers 4CL-GSP3 and 4CL-GSP4; a single *PgDHS2* genomic fragment was obtained with primers DHS2-GSP1 and DHS2-GSP2 (Supplementary Table S3B at *JXB* online). Amplified *Pg4CL* (GenBank JN828803) and *PgDHS2* (GenBank JN828804) genomic fragments (1885bp and 1353bp, respectively) were cloned using the TA Cloning Kit (Invitrogen) and electroporated into *Escherichia coli* XL1-Blue cells. Putative AC elements were identified with the Fuzznuc tool (http://mobyle.pasteur.fr/cgi-bin/portal.py; Néron *et al.*, 2009) using canonical AC motif sequences as search key words and in both orientations.

### Construction of promoter::GUS reporter and MYB expression vectors

Two overlapping genomic DNA fragments were then obtained with proximal primers 4CL-GSP1 and 4CL-GSP2, and distal primers 4CL-GSP3 and 4CL-GSP4; a single *PgDHS2* genomic fragment was obtained with primers DHS2-GSP1 and DHS2-GSP2 (Supplementary Table S3B at *JXB* online). The digested *Pg4CL* (*Xba*I/*Bam*HI) and *PgDHS2* (*Spe*I/*Bam*HI) fragments, 1885bp and 1353bp long, respectively, were inserted into a modified pMJM vector to create promoter::GUS (β-glucuronidase) fusions as described (Bedon *et al.*, 2009). They were then inserted into a modified pCAMBIA2300 vector where the hygromycin resistance gene was replaced by the silencing inhibitor p19 gene (Voinnet *et al.*, 2003). The MYB expression vectors were obtained by PCR amplification of the cDNA coding sequences of *PgMYB8* and *PgMYB15* as well as *PtMYB1*, *PtMYB8*, and *PtMYB14*. They were transferred into the pCAMBIA2300 expression vector using the Gateway^®^ system (Invitrogen). The *Pg/PtMYB* coding sequences were flanked by the *ubiquitin* promoter and the 35S terminator. Both reporter and expression vectors were transferred into *Agrobacterium tumefaciens* strain AGL1.

### Transactivation assay

A transient transactivation assay system was developed using *P. glauca* somatic embryogenic cells co-transformed with *Agrobacterium*, based on the stable transformation procedures previously described (Klimaszewska *et al.*, 2004). Briefly, the two *Agrobacterium* cultures (reporter and expression vectors) were incubated overnight, diluted to an optical density of 1.0, and used for co-cultivation with embryogenic cells (1h at 21 °C). The cells were separated from *Agrobacterium* by filtration, dispensed onto filter paper discs wetted with liquid medium supplemented with 50 μM acetosyringone, and kept in the dark at 21 °C. Four filter papers (replicate) were used per reporter–expression vector combination. After 6 d of co-culture, histochemical GUS assay was performed, and each GUS-stained filter was photographed separately. Images were analysed using the Assess 2.0 software (St Paul, MN, USA) to obtain a uniform determination (resulting densitometry) of GUS-positive cell clusters representing the maximum number of visible spots.

### Recombinant protein purification

For the *PgMYB8* expression plasmid, a synthetic DNA segment (GenScript, Piscataway, NJ, USA) was cloned into the pET300/NT-DEST vector (Invitrogen) using the Gateway^®^ technology. The *PgMYB14* and *PgMYB15* coding sequences were amplified by PCR with *Nde*I and *Xho*I primers specific to each gene. Each PCR product was ligated into *Nde*I and *Xho*I restriction sites in the pET-30a (+) vector (Novagen) and expressed in *E. coli* Rosetta strain (Novagen). At 4h after induction with 0.1mM isopropyl-β-d-thiogalactopyranoside, the culture was harvested by centrifugation for 5min at 5000 *g*. The pellet was resuspended in 50ml of ice-cold MBP buffer [10mM TRIS-HCl, pH 7.5, 30mM NaCl, 1mM EDTA, 1mM phenylmethylsulphonyl fluoride (PMSF), and 1× protease inhibitor cocktail], sonicated for 15min, and centrifuged for 20min at 14 000 *g*. The recombinant proteins were purified under denaturing conditions based on Haneskog (2006). Protein purification was verified by SDS–PAGE and Coomassie blue staining.

### Electrophoretic mobility shift assay (EMSA)

Purified PgMYB8, PgMYB14, and PgMYB15 recombinant proteins were tested for their ability to bind to oligonucleotides from the *Pg4CL* and *PgDHS2* promoters and containing specific AC elements by using EMSA. The 30bp fragments of *Pg4CL* and *PgDHS2* promoters used as probes were as follows: *4CL* AC-I, *4CL* AC-II, *4CL* mAC-I, and *4CL* mAC-II; and *DHS2* AC-I, *DHS2* AC-II, *DHS2* mAC-I, and *DHS2* mAC-II (Supplementary Table S3D at *JXB* online). The double-stranded oligonucleotides were end labelled with γ-^32^P. Recombinant protein (375ng) and labelled probe (0.2ng) were incubated at room temperature for 30min, with 100ng of poly(dI–dC) used as an unspecific competitor in a 20 μl reaction with a final concentration of 20mM TRIS pH 8.0, 10mM NaCl, 2mM EDTA, 2mM dithiothreitol (DTT), and 10% glycerol (v/v). Competitions used unlabelled double-stranded probes (20- or 200-fold excess). Electrophoreses of DNA–protein complexes were on 6% native polyacrylamide gels (1× TRIS-glycine buffer, 35 mA, 4 °C). Migration of radiolabelled probes and complexes was detected on Kodak Biomax XAR film.

DNA binding site competition analysis for the AC-I-containing probe from the *Pg4CL* promoter was also performed between PgMYB8 and PgMYB15 recombinant proteins. For this, preliminary experiments were performed to optimize probe and protein concentration. The molecular weight of PgMYB8 and PgMYB15 was determined using a 10% denaturing SDS–polyacrylamide gel and pre-stained protein marker (New England BioLabs). Protein concentration was calculated with Quick Start™ Bradford Protein Assay (Bio-Rad). Two types of competition experiments were performed by modifying the stoichiometric ratio between PgMYB8 and PgMYB15: (i) 0.7-, 1.0-, and 1.3-fold amounts of PgMYB8 were used to out-compete a constant amount of PgMYB15; while (ii) 20-, 40-, and 60-fold amounts of PgMYB15 were used to out-compete a constant amount of PgMYB8. To ascertain the competition effect, the concentration of PgMYB8 used in experiment (ii) was 10-fold less than in experiment (i). EMSA conditions were as previously described, except that the AC-I-containing probe concentration was 25- and 10-fold diluted for experiment (i) and (ii), respectively.

## Results

### Identification and annotation of co-expressed transcripts in spruce plants overexpressing pine *MYB* genes

**Fig. 1.**
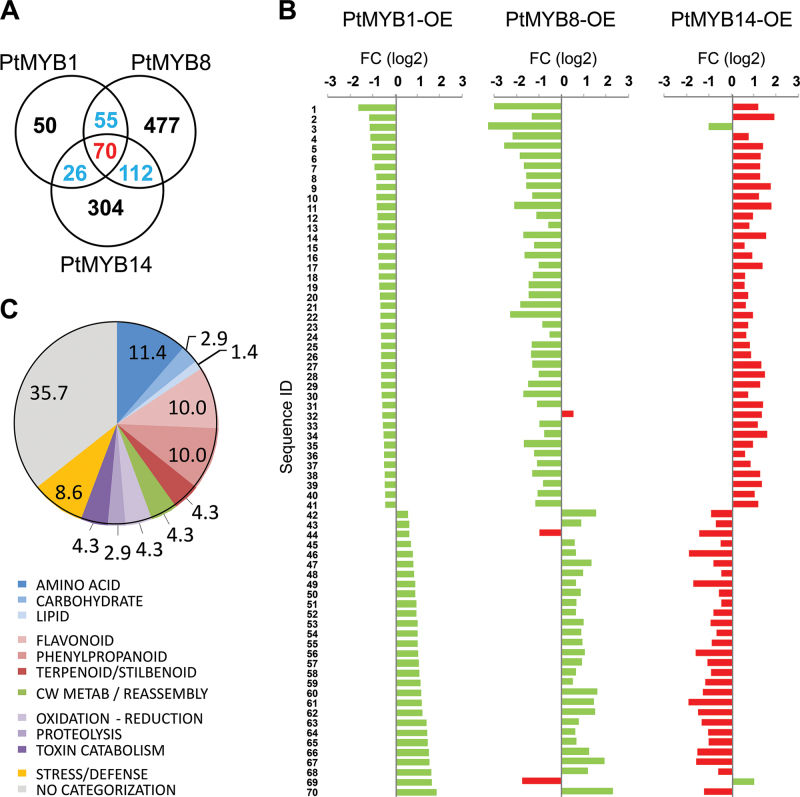
Comparative analysis of microarray profiles from *Pinus taeda* PtMYB1, PtMYB8, and PtMYB14 overexpression (OE) in spruce. (A) Numbers of shared and unique misregulated sequences in PtMYB1, PtMYB8, and PtMYB14OE. (B) Expression fold change (FC) of the 70 common misregulated sequences compared with the wild type. Red bars: opposite FC to MYB1-OE. See Table S2 for sequence IDs. (C) KEGG functional categories (%) of the 70 common sequences misexpressed in all three transgenic lines. For details, see Supplementary Table S1 at *JXB* online.

Analysis of microarray transcript profiles of transgenic spruce overexpressing the *P. taeda* genes *PtMYB1*, *PtMYB8* (Bomal *et al.*, 2008), and *PtMYB14* (Bedon *et al.*, 2010) identified 202, 715, and 513 misregulated sequences relative to controls, respectively (Fig. 1A; Supplementary Table S1 at *JXB* online). A total of 70 different sequences were misregulated in all three transgenic backgrounds (Fig. 1A), and nearly all of the transcripts (66 out of 70) showed the same pattern of accumulation in PtMYB1 and PtMYB8 transgenics but had opposite expression in PtMYB14 transgenics (Fig. 1B). A similar trend was observed for sets of misregulated genes obtained from pair-wise comparisons between PtMYB1 and PtMYB8 (55 transcripts), PtMYB1 and PtMYB14 (26 transcripts), and PtMYB8 and PtMYB14 overexpressors (112 transcripts) (Supplementary Fig. S1); an opposite misregulation was largely observed when comparing PtMYB14-overexpressing lines with PtMYB1 or PtMYB8-overexpressing lines, while a similar pattern of accumulation was observed in a PtMYB1 and PtMYB8 overexpressor comparison.

The set of 66 co-expressed sequences (with opposite expression) common to PtMYB1, PtMYB8, and PtMYB14 transgenics were linked to specific aspects of primary and secondary metabolism (Fig. 1C). The secondary metabolism sequences represented nearly 25% of the total and were linked to flavonoid (10%, PATH ath00941), phenylpropanoid (10%, PATH ath00940), and terpenoid biosynthetic pathways (4.3%, PATH ath00903) (Fig. 1C; Supplementary Table S2 at *JXB* online). Primary metabolism sequences (15.7% of the total) were distributed among amino acid (11.4%), carbohydrate (2.9%), and lipid (1.4%) metabolism. Sequences assigned to amino acid metabolism were related to cysteine and methionine metabolism (PATH ath00270), as well as phenylalanine, tyrosine, and tryptophan metabolism (PATH ath00400) (Supplementary Table S2). The remaining annotated sequences were assigned to processes such as oxidation–reduction, proteolysis and toxin catabolism (11.5%), cell wall metabolism (4.3%), and stress/defence (8.6%). Around a third of the sequences could not be linked to any known biological process. The sequences obtained from pair-wise comparisons gave similar functional categorizations, although this latter set had slightly higher proportions of sequences among biological processes of cell wall metabolism and stress or defence.

Overall, the sequences assigned to terpenoid and flavonoid pathways, cell wall relaxation, and stress or defence responses were preferentially up-regulated in transgenics overexpressing PtMYB14, consistent with its proposed role in defence (Bedon *et al.*, 2010). Up-regulation of transcripts associated with *S*-adenosyl methionine, shikimate, and phenylpropanoid biosynthetic pathways was observed in transgenics overexpressing PtMYB1 and PtMYB8 (Supplementary Table S2 at *JXB* online), consistent with their proposed structural roles including secondary cell wall assembly (Bomal *et al.*, 2008).

### Spatio-temporal transcript variation reveals positive and negative correlations among co-expressed genes and potential MYB regulators

Results obtained from the transgenic microarray profiles (Fig. 1; Supplementary Fig. S1 and Tables S1, S2 at *JXB* online) led to the hypothesis that these different conifer MYBs could target these groups of co-expressed genes in a competitive or antagonistic manner. Aiming to gather data in support of this hypothesis, transcript levels of the candidate target genes were monitored over a diurnal cycle in wild-type spruce trees. A diurnal variation for PgMYBs and co-expressed transcripts was expected based on reports for genes related to phenylpropanoid metabolism (Harmer *et al.*, 2000) and stress (Walley *et al.*, 2007). Reliable transcript accumulation results were obtained for 33 candidate gene sequences by quantitative PCR analysis (Supplementary Table S4). Spearman correlation rank tests were used to identify significant correlations (adjusted *P*-value ≤0.05, FDR of 5%) between the transcript levels of the candidate genes and those of *PgMYB1*, *PgMYB8*, and *PgMYB14* as well as *PgMYB15*, a close homologue of *PgMYB14* (Bedon *et al.*, 2010) (Table 1; Supplementary Tables S5, S6).

**Table 1.** ρ estimate from Spearman’s correlation rank test between expression data of PgMYB and co-expressed target sequences during a diurnal cycle in wild-type spruce

Sequence annotation and categorization			Xylem				Bark/phloem			
Name (PN)	GenBank	Path	MYB1	MYB8	MYB14	MYB15	MYB1	MYB8	MYB14	MYB15
Candidate target										
*SHM4 (49)*	BT117395	AA	0.32*^a^	0.39**	–0.15	–0.26*	0.29*	0.33**	–0.13	0.10
*MTFR2* (63)	BT119867	AA	0.14	0.30*	0.10	0.08	0.05	0.19	–0.12	0.16
*MEE58* (54)	BT106824	AA/SAM	0.48**	0.40**	–0.12	–0.12	0.54**	0.35**	0.03	0.17
*ADT2* (17)	BT102177	AA/SHI	0.18	0.02	–0.03	0.07	0.01	–0.04	–0.02	0.01
*CM1* (48)	BT115945	AA/SHI	0.39**	0.40**	–0.09	–0.25	0.35**	0.45**	–0.02	0.09
*DHS2 (67)*	BT116706	AA/SHI	0.43**	0.52**	–0.17	–0.35**	0.43**	0.61**	0.04	–0.04
*DFR* (10)	BT115327	FLA	–0.01	0.03	0.22	0.33*	0.09	0.19	0.03	–0.10
*CHI* (12)	BT101304	FLA	–0.24	–0.18	0.01	0.21	0.18	0.12	–0.10	–0.01
*LDOX* (16)	BT102953	FLA	–0.07	–0.05	0.19	0.34**	0.00	0.13	–0.19	–0.07
*TT4/CHS* (19)	BT103070	FLA	0.09	0.04	0.31*	0.39**	0.22	0.13	0.17	0.07
*COMT1 (42)*	*GQ0043_N14*	PHE	0.38**	0.59**	–0.08	–0.39**	0.50**	0.63**	0.13	0.21
*BGLU40* (5)	BT101848	PHE	0.18	0.08	0.29*	0.38**	–0.18	–0.26	0.14	–0.05
*HCT* (56)	BT117023	PHE	0.38**	0.52**	0.00	–0.04	0.45**	0.41**	0.03	0.09
*4CL2 (61)*	BT106671	PHE	0.34**	0.34**	–0.21	–0.58**	0.36**	0.38**	0.02	–0.04
*PRR2* (68)	BT111350	PHE	0.32*	0.32*	–0.19	–0.11	0.52**	0.60**	0.00	0.04
*CYP76G1* (11)	*GQ0205_N10*	TER	0.13	0.12	0.33**	0.42**	0.40**	0.36**	0.14	0.06
*XTH9* (1)	BT102510	CW	0.13	0.10	0.37**	0.38**	–0.12	–0.13	0.05	–0.07
*GH17* (38)	BT118546	CW	0.02	0.10	0.05	0.08	0.15	0.21	0.09	0.20
*CYP76C4* (27)	*GQ0133_I20*	OX	0.18	0.02	0.17	0.22	0.02	–0.12	0.13	0.08
*CYP76C5* (28)	BT116913	OX	0.22	0.28*	0.04	0.15	0.00	0.13	0.12	0.03
*GSTU18* (33)	BT103013	TOX	0.01	–0.24	–0.01	0.37**	0.09	0.23	0.18	0.22
*Lipase/thio (66)*	BT102121		0.37**	0.53**	–0.29*	–0.40**	0.43**	0.46**	–0.07	0.08
*HRGP* (3)	BT117438		–0.06	–0.10	0.29*	0.37**	0.07	0.02	–0.14	0.17
No hit (20)	BT112709		–0.07	–0.20	0.09	0.29*	–0.07	–0.06	0.12	0.01
No hit (22)	BT113910		–0.13	–0.08	0.12	0.49**	0.20	0.16	0.19	0.25
*DAG* (37)	BT115660		0.07	0.22	–0.22	–0.12	0.17	–0.02	–0.08	0.07
*EDGP* (40)	BT115833		–0.05	0.08	0.24	0.20	0.21	0.31**	0.08	–0.10
*PR4* (44)	BT117075		0.12	0.03	–0.03	–0.16	0.32**	0.07	0.11	0.13
*KT2* (45)	BT116079		0.31*	0.56**	–0.17	0.04	0.13	0.20	–0.07	0.26
*HB3* (51)	BT102955		0.15	0.04	0.21	0.13	0.60**	0.47**	0.28*	0.00
*LTP* (52)	BT103567		0.02	0.05	0.08	0.28*	0.18	0.28*	0.00	0.19
*Cys Pase* (55)	BT114584		0.39**	0.33**	0.06	0.02	0.16	0.23	0.05	0.14
*ATAF1* (58)	BT115770		0.18	0.20	0.13	0.11	0.17	0.07	0.16	0.10
MYB TF										
*MYB1*	BT108631		1				1			
*MYB8*	BT108136		0.43**	1			0.59**	1		
*MYB14*	FJ469917		0.03	0.00	1		0.25*	0.08	1	
*MYB15*	FJ469918		–0.23	–0.24	0.37**	1	0.15	0.20	0.09	1

^*a*^ ρ estimates in bold were significant at *P*-values ≤0.05 (*) or ≤0.01(**) in a two-tailed Spearman test. Due to multiple comparison tests, a *q*-value was calculated to estimate a minimum FDR to adjust the significance (*P*-value) of ρ estimates.

Genes positively correlated with PgMYB1/PgMYB8 and negatively correlated with PgMYB14/PgMYB15 are underlined.

Complete expression data sets from the diurnal cycle experiment and used for Spearman’s correlation rank tests are presented in Supplementary Table S4 at *JXB* online. PN, probe number. Path: CW, cell wall; PHE, phenylpropanoid; FLA, flavonoid; TER, terpenoid; AA, amino acid; SHI, shikimate; SAM, *S*-adenosylmethionine; OX, oxidation–reduction; TOX, toxin catabolism.

Complete correlation tests are presented in Supplementary Table S5 (xylem) and Table S6 (bark/phloem).

The transcripts of five genes correlated positively (adjusted *P*-value ≤0.034, *q-*value ≤0.05) with those of *PgMYB1* and *PgMYB8*, and negatively with those of *PgMYB15* and, to a lesser extent, *PgMYB14* (see Table 1). These genes were annotated as coding for 4-coumarate CoA ligase (4CL), 3-deoxy-7-phosphoheptulonate synthase (DHS2), caffeic acid *O*-3-methyltransferase 1 (COMT1), serine hydroxymethyltransferase 4 (SHM4), and a lipase/thioesterase (Lipase/thio). This transcript profiling experiment was carried out in actively growing secondary tissues of xylem and in bark with phloem. The opposite correlations were observed only in the xylem and not in the bark/phloem (Table 1). As they were obtained from a group of plants of diverse genetic backgrounds and from several sampling time points, the correlations may be relatively robust.

Transcripts from several other sequences gave significant positive correlations (ρ estimates) (Table 1) that were consistent with observations in the transgenic spruce plants, but nearly all of the significant correlations were uniquely observed with those of *PgMYB1* and *PgMYB8*, or uniquely with *PgMYB15* and *PgMYB14*. The transcript levels of *PgMYB1* and *PgMYB8* were positively correlated in both xylem and bark/phloem, with sequences assigned to phenylpropanoid (HCT and PRR2), shikimate (CM1), and *S*-adenosylmethionine (MEE58) metabolism, and with sequences not assigned to a particular metabolic process, such as KT2 for xylem and CYP76G1 and HB3 for bark/phloem tissues (Table 1). The observed negative correlations with transcripts of *PgMYB1* and *PgMYB8* were very weak (non-significant) in both tissue types. In contrast, the positive correlations with transcripts of *PgMYB15* and *PgMYB14* (to a lesser extent) were only observed in xylem and not in the bark/phloem tissues. These sequences were related to flavonoid (DFR, LDOX, and TT4/CHS), terpenoid (CYP76G1), phenylpropanoid (BGLU40), and cell wall (XTH9) metabolism, as well as toxin catabolism (GST18).

Analyses of transcript accumulation data were expanded to include 10 additional PgMYBs, putatively linked to secondary cell wall deposition and stress response (Bomal *et al.*, 2008; Bedon *et al.*, 2010). Transcript accumulation of the target genes that significantly correlated with *PgMYB1*, *PgMYB8*, *PgMYB14,* and *PgMYB15* also correlated with several other *PgMYB* genes (Supplementary Tables S5, S6 at *JXB* online): significant correlation estimates were obtained between expression data of *PgMYB1*/*PgMYB8* and those of *PgMYB2*, *PgMYB4*, *PgMYB16*, and *PgMYB17* in xylem and bark/phloem tissues, while expression data of *PgMYB14*/*PgMYB15* correlated with those of *PgMYB13* in xylem tissues, and *PgMYB3*, *PgMYB5*, and *PgMYB13* in bark/phloem tissues.

### Transactivation assays reveal opposite effects of selected spruce and pine MYBs on candidate target promoters

The transactivation and repression of promoters from two of the candidate target genes (*Pg4CL* and *PgDHS2*) were tested for the four different MYBs hypothesized to have opposite effects on expression (see Materials and methods). Upstream flanking sequences were isolated for *Pg4CL* (1885bp) and *PgDHS2* (1353bp), and assayed functionally for gene expression activity by co-transformation in *P. glauca* cell cultures. Strong evidence for transactivation of the *Pg4CL* and *PgDHS2* promoters was obtained for the spruce gene *PgMYB8* and for the pine genes *PtMYB1* and *PtMYB8* (Table 2). For each of the combinations tested, the GUS activity stain increased from 3- to ≥10-fold, compared with the controls transformed with the promoter construct alone. In contrast, co-transformation with either the spruce gene *PgMYB15* or the pine gene *PtMYB14* decreased the GUS staining to ≤20% of the level observed in the controls, representing at least a 5-fold decrease (Table 2).

**Table 2.** Densitometry analysis of GUS staining in transactivation assays for MYB–promoter interactions

TF vector	GS^*a*^	*PgDHS2*pro			*Pg4CL*pro		
		No TF^*b*^	TF^*c*^	Ratio TF/no TF	No TF	TF	Ratio TF/no TF
*PgMYB8*	Spot	24	223	**9.29**	26	308	**11.85**
	Density	3254	34 590	**10.63**	3574	45 939	**12.85**
*PgMYB15*	Spot	24	2	**0.08**	26	0	**0.00**
	Density	2291	266	**0.12**	3574	0	**0.00**
*PtMYB1*	Spot	113	373	**3.30**	16	85	**5.31**
	Density	17 298	63 295	**3.66**	2424	14 279	**5.89**
*PtMYB8*	Spot	72	191	**2.65**	28	156	**5.57**
	Density	9672	31 545	**3.26**	3347	18 595	**5.56**
*PtMYB14*	Spot	168	36	**0.21**	75	11	**0.15**
	Density	24347	4858	**0.20**	11258	1397	**0.12**

^*a*^ GUS staining (GS), number of blue spots or total density across all four filters (replicates) (see Materials and methods).

^*b*^ No TF, empty vector control.

^*c*^ TF, MYB expression vector.

### PgMYB8 and PgMYB15 bind to and compete for AC *cis*-elements present in both *Pg4CL* and *PgDHS2* promoters

**Fig. 2:**
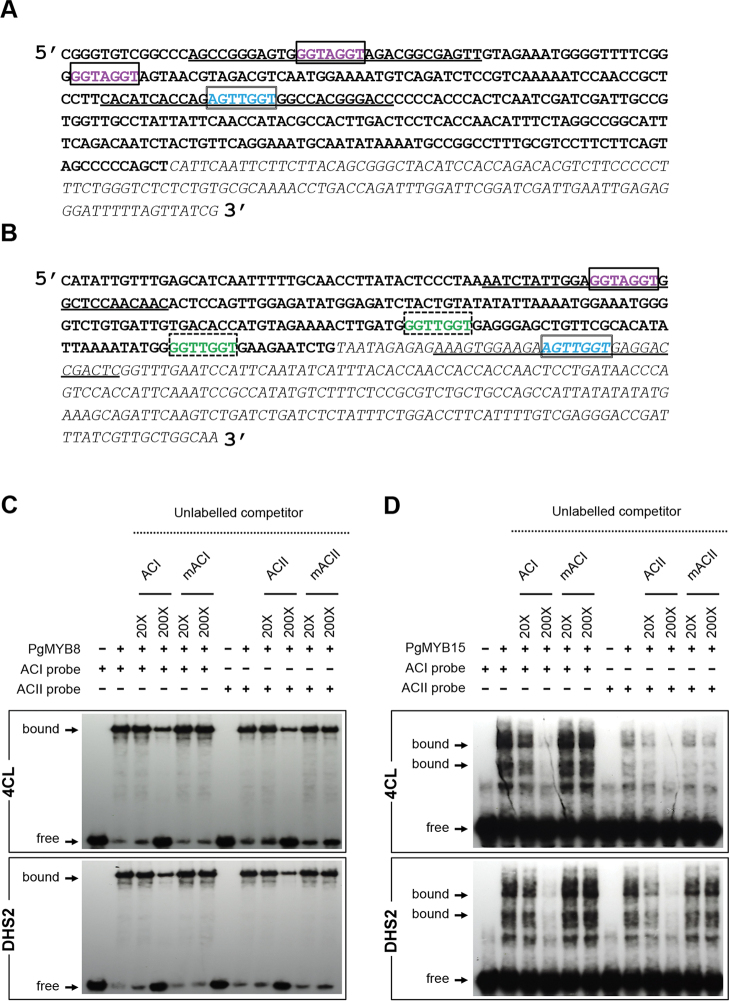
Analysis of PgMYB8 and PgMYB15 binding to AC elements present in *Pg4CL* and *PgDHS2* promoters in spruce. (A, B) Upstream flanking and 5′ UTR sequences (450bp) of the spruce (A) *Pg4CL* and (B) *PgDHS2* genes. For both *Pg4CL* and *PgDHS2* sequences, underlined nucleotides correspond to the 30bp probes (containing either an AC-I or AC-II element) designed for EMSA with PgMYB8, PgMYB14, and PgMYB15 recombinant proteins. The sequences shown represent the regions in which AC elements were identified in the *4CL* promoter (1885bp) and *DHS2* promoter (1353bp). Nucleotide representations are as follows: bold in upstream regions; italics in 5′ UTR sequences; AC-I sequences are boxed and AC-II sequences have a double box (ACCAACT) or a dashed box (ACCAACC). (C, D) EMSA testing of the binding of PgMYB8 (C) and PgMYB15 (D) recombinant protein to the labelled AC-I- and AC-II-containing probe [30bp fragments from *Pg4CL* (top) and *PgDHS2* (bottom) promoters; see Fig. 3 for details]. Competition analyses used unlabelled probes containing AC-I, ACII, or mutated AC elements (mAC). Electrophoretic shifts (bound) and free DNA probes are indicated by arrows. (This figure is available in colour at *JXB* online.)

Promoters of several genes of the monolignol and shikimate biosynthetic pathways have been shown to contain AC elements (Patzlaff *et al.*, 2003*a*, *b*; Raes *et al.*, 2003; Chen *et al.*, 2006; Prouse and Campbell, 2012). Both AC-I (ACCTACC) and AC-II (ACCAAC^C^/_T_) sequences were found to be present in the upstream flanking and 5′-untranslated region (UTR) sequences of the *Pg4CL* and *PgDHS2* spruce genes (Fig. 2A, B). An AC-I sequence matching the canonical ACCTACC was found in one and two copies in 5′ upstream flanking sequences of *PgDHS2* and *Pg4CL* genes, respectively; one putative AC-I box from each promoter was targeted for binding assays (Fig. 2A, B). For AC-II, one putative element was located in the 5′ upstream region of the *Pg4CL* gene (ACCAACT), whereas two copies of a different sequence (ACCAACC) were 5′ upstream of the *PgDHS2* gene, and one copy of ACCAACT was in the 5′ UTR (Fig. 2A, B). Aiming to compare the interaction of different MYBs and an AC-II element associated with each gene directly, the conserved sequence (ACCAACT) was targeted in the oligonucleotides despite the location of one of them in a 5′ UTR. To examine whether PgMYB8, and PgMYB15 were able to bind to these putative *cis*-elements, 30bp oligonucleotide probes (containing AC-I or AC-II elements and flanking regions of either *Pg4CL* or *PgDHS2* promoters; Fig. 2C, D) were used in EMSAs.

PgMYB8 and PgMYB15 recombinant proteins were able to bind specifically the AC-I element present in the promoter region of *Pg4CL* and *PgDHS2* genes. A clear mobility shift was obtained for the AC-I-containing probe in the presence of PgMYB8 (Fig. 2C) as well as PgMYB15 (Fig. 2D). Unlabelled DNA oligonucleotides containing AC-I *cis*-elements in increasing concentrations competed with the binding of PgMYB8 and PgMYB15, while those containing a mutated AC element did not. The EMSA performed with the AC-II elements from both of the promoters indicated a clear mobility shift for PgMYB8 (Fig. 2C). In contrast, weak affinity for the AC-II element in the *DHS2* probe was observed with PgMYB15 (Fig. 2D). For PgMYB8 and PgMYB15, competition with unlabelled AC-II probe altered the binding profile, while a AC-II mutated probe did not compete (Fig. 2C, D). No evidence for mobility shift was seen with a *Pg4CL* AC-II-containing probe and recombinant protein PgMYB15 (Fig. 2D).

**Fig. 3.**
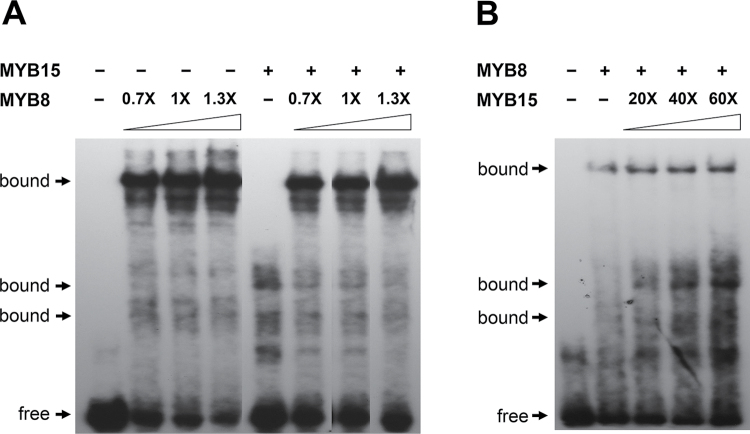
DNA binding competition analysis between PgMYB8 and PgMYB15 for an AC-I-containing promoter fragment from *Pg4CL*. EMSA using MYB8 and MYB15 recombinant proteins and labelled oligonucleotides from *Pg4CL* with a variable amount of (A) PgMYB8 protein or (B) PgMYB15 protein. Increasing stoichiometric ratios of MYB recombinant protein are indicated. Electrophoretic shifts (bound) and free DNA probes are indicated by arrows. Exposure showing the best resolution of the specific TF–DNA complex was used for each panel.

DNA binding site competition analysis for the AC-I-containing probe from *Pg4CL* indicated that PgMYB8 recombinant protein had a very strong affinity for the AC-I element compared with PgMYB15 (Fig. 3). PgMYB8 effectively out-competed PgMYB15 at a stoichiometric ratio of 0.7 (PgMYB8 to PgMYB15) (Fig. 3A), while a 60-fold amount of PgMYB15 was inefficient to out-compete a constant amount of PgMYB8 (Fig. 3B).

## Discussion

Results from the present study identified a set of target genes encoding enzymes of primary and secondary metabolism that are proposed to be differentially regulated by the two types of R2R3-MYBs in *P. glauca*. Several of the pathways are interconnected, and flux into each one of them may vary during development or in response to environmental cues. Evidence has been provided that regulation of these genes may involve complex transcriptional control by different MYBs with opposite effects, as well as MYBs with overlapping functions.

### Discovery of putative common target genes for conifer MYBs belonging to different subgroups

This report presents comparative analyses of two pairs of functionally redundant conifer R2R3-MYBs from pine (Pt) and spruce (Pg), namely Pt/PgMYB1 and Pt/PgMYB8 of subgroups SG8 and SG13 (Bedon *et al.*, 2007), and Pt/PgMYB14 and Pt/PgMYB15 of subgroup SG4 (Bedon *et al.*, 2010), which were shown to impact different aspects of primary and secondary metabolism. On the one hand, Pt/PgMYB1 and Pt/PgMYB8 were linked to positive control of shikimate and monolignol biosynthetic genes and were proposed to be members of a transcriptional network similar to the SND1 cascade in spruce and pine (Bomal *et al.*, 2008), which regulates secondary cell wall formation in *Arabidopsis* (Zhong *et al.*, 2007, 2010). On the other hand, Pt/PgMYB14 and Pt/PgMYB15 were identified as putative positive regulators of isoprenoid and flavonoid metabolism, and linked to the regulation of defence responses in spruce and pine (Bedon *et al.*, 2010).

In the present study, a comparative analysis of transcript profiles from constitutive overexpression of PtMYB1, PtMYB8, and PtMYB14 in spruce indicated that MYBs from different subgroups (SG8, SG13, and SG4) may impact a common set of genes. The transcript profiling results also pointed to activation or repression (i.e. up-/down-regulation) depending on the MYB that is overexpressed. Specifically, genes that were co-expressed in PtMYB1- and PtMYB8-overexpressing lines (e.g. up-regulation of shikimate and monolignol genes and down-regulation of flavonoid genes) had opposite expression in PtMYB14-overexpressing lines (Fig. 1). The fact that 95% of the common sequences displayed an opposite expression profile between PtMYB1/PtMYB8 and PtMYB14 (Fig. 1) is unlikely to appear by chance. In addition, pair-wise comparisons between PtMYB1 and PtMYB14, or PtMYB8 and PtMYB14 gave opposite expression profiles for 95% and 89% of the genes, respectively (Supplementary Fig. S1 at *JXB* online).

It has been shown that constitutive overexpression of TFs may cause pleiotropic effects (Zhang, 2003). In this context, the constitutive accumulation of PtMYBs in transgenic spruce may have sequestrated components of the transcriptional machinery away from *cis-*regulatory DNA elements by competing with cognate TFs (Gill and Ptashne, 1988). It was observed that the spatio-temporal transcript profiles of *PgMYB* genes and some of the candidate target genes were poorly correlated during a diurnal cycle, whatever the tissues tested (Table 1), indicating that pleiotropic effects may have affected some genes. Nevertheless, the RNA transcript profiling results were consistent with the proposed opposition effect, in a time-related and tissue-preferential manner, for several sequences in non-transgenic spruce plantlets (Table 1; Supplementary Tables S4, S5 at *JXB* online). Together, the expression profiles from the transgenics and the diurnal profile suggested that different conifer MYBs (from SG8, SG13, and SG4 subgroups) could directly target genes coding for key enzymes from interconnected pathways of amino acid (DHS2 and SHM4) and phenylpropanoid (4CL and COMT1) metabolism, specifically in differentiating secondary xylem. Another explanation for the identification of a set of common misregulated genes could be linked to indirect effects of PtMYB overexpression, such as non-specific binding to *cis*-elements by highly conserved DBDs, as observed among conifer MYBs (Bedon *et al.*, 2007). As suggested in *Arabidopsis* (Jin *et al.*, 2000), structural genes with similar *cis-*regulatory motifs may be strongly influenced by such a dosage-linked effect and may become coordinately regulated due to high levels of specific regulators. This is likely to appear in gain-of-function experiments where abnormally high levels of MYBs are induced by overexpression. In the transgenic spruce used here, *PtMYB1*, *PtMYB8*, and *PtMYB14* transcript levels represented 33-, 40-, and 300-fold increases compared with their spruce endogenous counterparts *PgMYB1*, *PgMYB8*, and *PgMYB14*, respectively (Bomal *et al.*, 2008; Bedon *et al.*, 2010). However, these latter *PgMYB* transcripts showed a 3- to 5-fold variation in non-transgenic plants during the diurnal cycle. Under such circumstances, unspecific binding due to high levels of MYBs would be unexpected.

### Competition between MYBs from different subgroups for the regulation of shikimate and phenylpropanoid biosynthesis genes in conifers

It has been shown here that conifer R2R3-MYBs from different subgroups could have direct and opposite effects on the regulation of two pivotal genes encoding enzymes of the shikimate and monolignol biosynthetic pathways: *DHS2* (3-deoxy-d-arabino-heptulosonate 7-phosphate synthase), which provides precursors for phenylpropanoid and flavonoid metabolism (Amthor, 2003), and *4CL (*4-coumarate:CoA ligase), which converts 4-coumaric acid and other substituted cinnamic acids for use in the biosynthesis of phenolic compounds and the production of flavonoids and monolignols (Ehlting *et al.*, 1999; Amthor, 2003). Transactivation assays and EMSAs provided two lines of evidence, in addition to expression data that support this conclusion. First, transactivation assays revealed strong activation of the *Pg4CL* and *PgDHS2* promoters for the spruce gene *PgMYB8* of SG13 and a repression effect of *PgMYB15* (SG4) on both promoters. The closest pine homologues (*PtMYB1*, *PtMYB8*, and *PtMYB14*) showed similar activation and repression effects. Secondly, PgMYB8 (SG13) as well as PgMYB14 and PgMYB15 (SG4) recombinant proteins are able to bind the same *cis*-element (AC-I) in the promoter region of these two genes. In *Arabidopsis*, typical AC *cis*-elements, also called MYB recognition elements (MREs) (Feldbrügge *et al.*, 1997), were found in the promoters of the majority of shikimate, flavonoid, and monolignol biosynthetic genes (Raes *et al.*, 2003; Hartmann *et al.*, 2005; Chen *et al.*, 2006; Prouse and Campbell, 2012).

Two types of AC elements, AC-I (ACCTACC) and AC-II (ACCAAC^C^/_T_), were identified in the promoter region of both the *PgDHS2* and *Pg4CL* genes in spruce. EMSA analyses revealed that PgMYB8 (SG13), PgMYB14 (SG4), and PgMYB15 (SG4) recombinant proteins are able to bind the AC-I element in both of the promoter environments tested, suggesting a lack of MYB subgroup specificity for this element. Conversely, the PgMYB8 recombinant protein showed a strong affinity for the AC-II element while PgMYB14 and PgMYB15 showed no or weak affinity, respectively. The position of AC-II elements varied between the two genes (either 5′ upstream flanking or in the putative 5′ UTR) but only had a small impact on the binding results. The extended affinity of conifer MYB for different AC elements (AC-I, AC-II, and AC-III) has been previously reported for PtMYB1 (Patzlaff *et al.*, 2003*a*, *b*) and PtMYB4 (Gómez-Maldonado *et al.*, 2004; Morse *et al.*, 2009). Interestingly, recent work in *Pinus pinaster* (Pp) has also shown that PpMYB8 could bind an AC-II element in the promoter region of the *Prephenate AminoTransferase* (*PAT*) gene from the arogenate pathway (Craven-Bartle *et al.*, 2013). Thus, the affinity of spruce PgMYB8, PgMYB14, and PgMYB15 for AC elements in promoters of other genes of shikimate, arogenate, and phenylalanine metabolism should be further explored and extended to PgMYB from different subgroups.

The idea that a single target gene may be under the control of functionally redundant MYBs from the same subgroup is supported by increasing evidence (see Dubos *et al.*, 2010; Zhong *et al.*, 2010). In *Arabidopsis*, AtMYB58 and AtMYB63 (both of the SG3 subgroup) directly activated phenylpropanoid biosynthetic genes from PAL to CAD (Zhou *et al.*, 2009). Similarly, AtMYB11, AtMYB12, and AtMYB111 (all of the SG7 subgroup) activated the transcription of target genes from *CHALCONE SYNTHASE* (CHS) to *FLAVONOL SYNTHASE* (FLS) genes from flavonoid biosynthetic pathways (Stracke *et al.*, 2007). Redundancy in repression has also been observed for *Antirrhinum* AmMYB308 and AmMYB330 for the genes between C4H and CAD from the phenylpropanoid and monolignol biosynthetic pathways (Tamagnone *et al.*, 1998). In the conifers spruce and pine, PgMYB1, PgMYB8, and PgMYB14/PgMYB15 are phylogenetically distinct but share conserved DBDs (Bedon *et al.*, 2007, 2010). Therefore, their ability to compete for *cis*-elements via conserved DBDs is not unexpected. The affinity for AC-I *cis*-elements observed for PgMYB8 and PgMYB14/PgMYB15 is congruent with this explanation and might be the basis of competition among MYBs on the same target. However, DNA binding site competition analysis revealed a strong propensity of PgMYB8 recombinant protein to bind the AC-I element when competing with PgMYB15 (Fig. 3). Although other methods (such as quantitative assay in yeast or surface plasmon resonance) should be used to ascertain this latter result fully, it suggests that particular tissular and/or environmental (nutrition, stress) conditions may be needed for PgMYB15 to compete more strongly with PgMYB8. A signature motif similar to the basic helix–loop–helix (bHLH) interaction site has been identified in PgMYB14 and PgMYB15 peptide sequences (Bedon *et al.*, 2010). The bHLH motif may entail interactions with other proteins, such as bHLH and WD40, which have been identified as important MYB partners in flavonoid pathways in different plant species (Vom Endt *et al.*, 2002; Ramsay and Glover, 2005). In addition, the conifer MYBs from the present study vary in their C-termini, which typically harbour the activation and repression domains (Dubos *et al.*, 2010). According to Bedon *et al.* (2010), PgMYB14 and PgMYB15 harbour the C2 repressor motif containing the core EAR motif defined by Kranz *et al.* (1998). Interestingly, the *E. grandiis EgMYB1* sequence was shown to be very close to Pt/Pg MYB14/15 (Bedon *et al.*, 2007, 2010) and was shown to act as a repressor of lignin biosynthesis (Legay *et al.*, 2010). The differential regulatory effect of these PgMYBs on the same target is likely to be due to the presence of activation or repression domains, but complementary functional studies are needed to demonstrate whether PgMYB14 and PgMYB15 may act as active or passive repressors.

### MYBs as competing activators and repressors within a single transcriptional network for the control of primary and secondary metabolism

Several R2R3-MYBs have been described as regulators of specific aspects of primary or secondary plant metabolism, such as flavonoid, anthocyanin, monolignol, benzenoid, isoprenoid, shikimate, or glucosinolate biosynthesis (see Dubos *et al.*, 2010; Feller *et al.*, 2011). Their role has generally been associated with one single pathway, with an activation or repression function within the transcriptional network. However, studies in maize and *Arabidopsis* pointed out the dual activation/repression effect of R2R3-MYBs on genes from different pathways that compete for carbon flux. *Zea mays* ZmMYB31 was overexpressed in *Arabidopsis* and shown to down-regulate several genes involved in monolignol biosynthesis but also to up-regulate some flavonoid biosynthesis genes (Fornalé *et al.*, 2010). The authors proposed that ZmMYB31 might play an important role in carbon partitioning between these pathways. Similarly, *Arabidopsis* AtMYB75 was shown to regulate anthocyanin biosynthesis positively in early development and to regulate the accumulation of lignin negatively in older plants (Bhargava *et al.*, 2010). A similar dual effect on monolignol and anthocyanin biosynthetic pathways was revealed in the *atmyb32* mutant that had an increased level of COMT1 transcripts and decreased amounts of DFR and ANS transcripts compared with the wild type (Preston *et al.*, 2004).

### Evolution of R2R3-MYBs and plant transcriptional networks

**Fig. 4.**
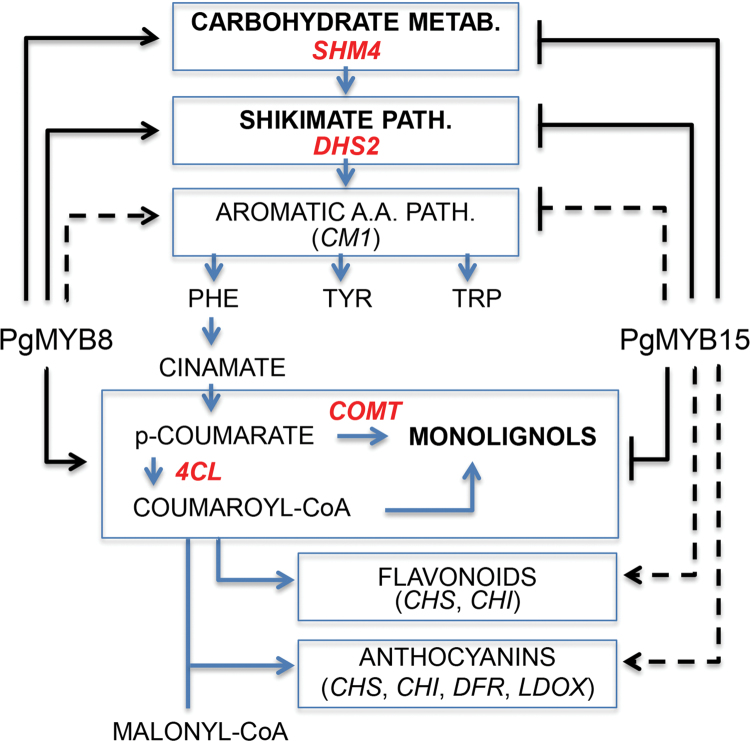
Proposed action of MYBs in the regulatory network controlling primary and secondary metabolism in conifers. An antagonistic action of PgMYB8 (activation) and PgMYB15 (repression) on interconnecting metabolic pathways is underlined (in bold): DHS2, 4CL, SHM4, and COMT are identified (boxed) as target genes under the control of both MYBs. Dashed lines indicate putative indirect effects of PgMYB15 on flavonoid and anthocyanin metabolic pathways. The network is also thought to involve PgMYB1 and PgMYB14, among others, which have not yet been tested in promoter binding assays but produce similar results as PgMYB8 (activation) and PgMYB15 (repression) in co-transformation assays, respectively. Genes that may fall under the antagonistic action of other MYBs are in parentheses. PHE, phenylalanine; TYR, tyrosine; TRP, tryptophan. (This figure is available in colour at *JXB* online.)

The present study identified conifer MYBs that could act on different aspects of primary and secondary metabolism by modulating the expression of key target genes from interconnecting pathways (Fig. 4). These MYBs belong to different subgroups and have opposite effects on two pivotal genes in the shikimate and monolignol biosynthetic pathways. As proposed in Fig. 4, PgMYB15 could thus impact carbon flux dedicated to monolignol synthesis by competing negatively for promoter binding sites with PgMYB8. This repression could simultanously affect gene expression of SHM4 (carbohydrate metabolism level), DHS2 (shikimate level), as well as 4CL and COMT (monolignol level). Due to its repressive action, PgMYB15 could indirectly stimulate flavonoid and/or anthocyanin metabolism. In addition, the co-expression patterns of several other MYBs tested in the system used here indicate that regulation in these pathways may be extended to include other MYBs as well (see Supplementary Tables S5, S6 at *JXB* online).

Evidence was previously obtained of partial redundancy among two R2R3-MYBs from *P. taeda*, PtMYB1 and PtMYB8, involved in monolignol regulation, and it was proposed that spruce and pine MYB8 could cross-regulate other R2R3-MYBs (Bomal *et al.*, 2008), but comparative data have been lacking. A genome-wide phylogenetic investigation of plants indicated that most families of TFs have expanded very significantly in flowering plants compared with Bryophyta/Lycopodiophyta, typically doubling in size around the time of their radiation (Lang *et al.*, 2010). Gymnosperms were not included in the study of Lang *et al.* (2010) but, as a representative of the gymnosperm lineage, *P. glauca* was estimated to have fewer MYBs than angiosperms: a total of 120 R2R3-MYBs, representing 0.43% of the catalogue of 27 720 unique expressed sequences (Rigault *et al.*, 2011), compared with 0.96% of the annotated genes for both *Arabidopsis* and poplar. However, at least one subfamily of R2R3-MYBs (e.g. the SG4 subgroup) was shown to be larger in conifers than in most angiosperms (Bedon *et al.*, 2010). More thorough investigations of the MYB family evolution in conifers to confirm their divergence with angiosperms may now be undertaken with the recent availability of the *P. glauca* genome (Birol *et al.*, 2013) and the *P. abies* genome (Nystedt *et al.*, 2013), although tracing their evolution will need a broader analysis. In turn, functional relationships between MYBs are likely to be closely linked to gene family evolution. The present findings in *P. glauca* R2R3-MYBs indicate that functional redundancy and antagonistic effects among R2R3-MYBs may be more widespread among seed-bearing plant taxa than previously shown.

In conclusion, it is proposed that MYBs from the present study are part of a transcriptional network covering complementary aspects of primary and secondary metabolism in conifer trees, as illustrated in Fig. 4. Within this network, MYBs may act as competing activators and/or repressors of key target genes from interconnecting biosynthetic pathways. This dual mode of action of MYB proteins may confer a way for plants to switch their metabolism in response to environmental cues, resulting in phenotypic plasticity. Gene duplication is considered as an important source of material for evolutionary novelties (Lynch and Conery, 2000) and has been evoked as an important mechanism leading to new functions for *R2R3-MYB* genes (Grotewold, 2005; Feller *et al.*, 2011). Monophyletic gene family amplification, suggested for conifer R2R3-MYB genes from subgroup SG4 (Bedon *et al.*, 2010), could be the basis of functional compensation needed to preserve the stoichiometric relationship between activators and repressors within a transcriptional network.

## Supplementary data

Supplementary data are available at *JXB* online.

Figure S1. Comparative analysis of microarray data associated with *Pinus taeda* MYB overexpression in spruce.

Table S1. Cross-comparison between 9K microarray expression data obtained from wild type (WT) versus PtMYB1, PtMYB8, and PtMYB14 overexpressors.

Table S2. Predicted annotations and KEGG functional categorization of co-expressed sequences common to PtMYB1, PtMYB8, and PtMYB14 overexpressors.

Table S3. Primer sequences used for quantitative PCR analysis, molecular cloning, and EMSAs.

Table S4. Transcript accumulation of *PgMYB*, and putative target genes in secondary xylem and bark/phloem tissues during a diurnal cycle of 3-year-old spruce.

Table S5. Spearman’s rank test correlation coefficients for pair-wise comparison between expression data of *PgMYB* and putative target genes in secondary xylem tissue of 3-year-old spruce.

Table S6. Spearman’s rank test correlation coefficients for pair-wise comparison between expression data of *PgMYB* and putative target genes in bark/phloem tissue of 3-year-old spruce.

Table S7. Densitometry data of GUS staining in transactivation assays for MYB–promoter interactions.

Supplementary Data
