# Supplementary material for: Opposite action of R2R3-MYBs from different subgroups on key genes of the shikimate and monolignol pathways in spruce
Source: J Exp Bot. 2013 Dec 14;65(2):495–508. doi: 10.1093/jxb/ert398 (PMC3904711; doi:10.1093/jxb/ert398)
Supplement: Supplementary Data [file supp_65_2_495__index.html]

Opposite action of R2R3-MYBs from different subgroups on key genes of the shikimate and monolignol pathways in spruce — Opposite action of R2R3-MYBs from different subgroups on key genes of the shikimate and monolignol pathways in spruce — Supplementary Data 

# Opposite action of R2R3-MYBs from different subgroups on key genes of the shikimate and monolignol pathways in spruce

## Supplementary Data

Data files

**Files in this Data Supplement:**

- Supplementary Data - Supplementary Data
- Supplementary Data - Supplementary Data
