# Supplementary material for: Opposite action of R2R3-MYBs from different subgroups on key genes of the shikimate and monolignol pathways in spruce
Source: J Exp Bot. 2013 Dec 14;65(2):495–508. doi: 10.1093/jxb/ert398 (PMC3904711; doi:10.1093/jxb/ert398)
Supplement: Supplementary Data [file supp_ert398_jexbot105460_file002.pdf]

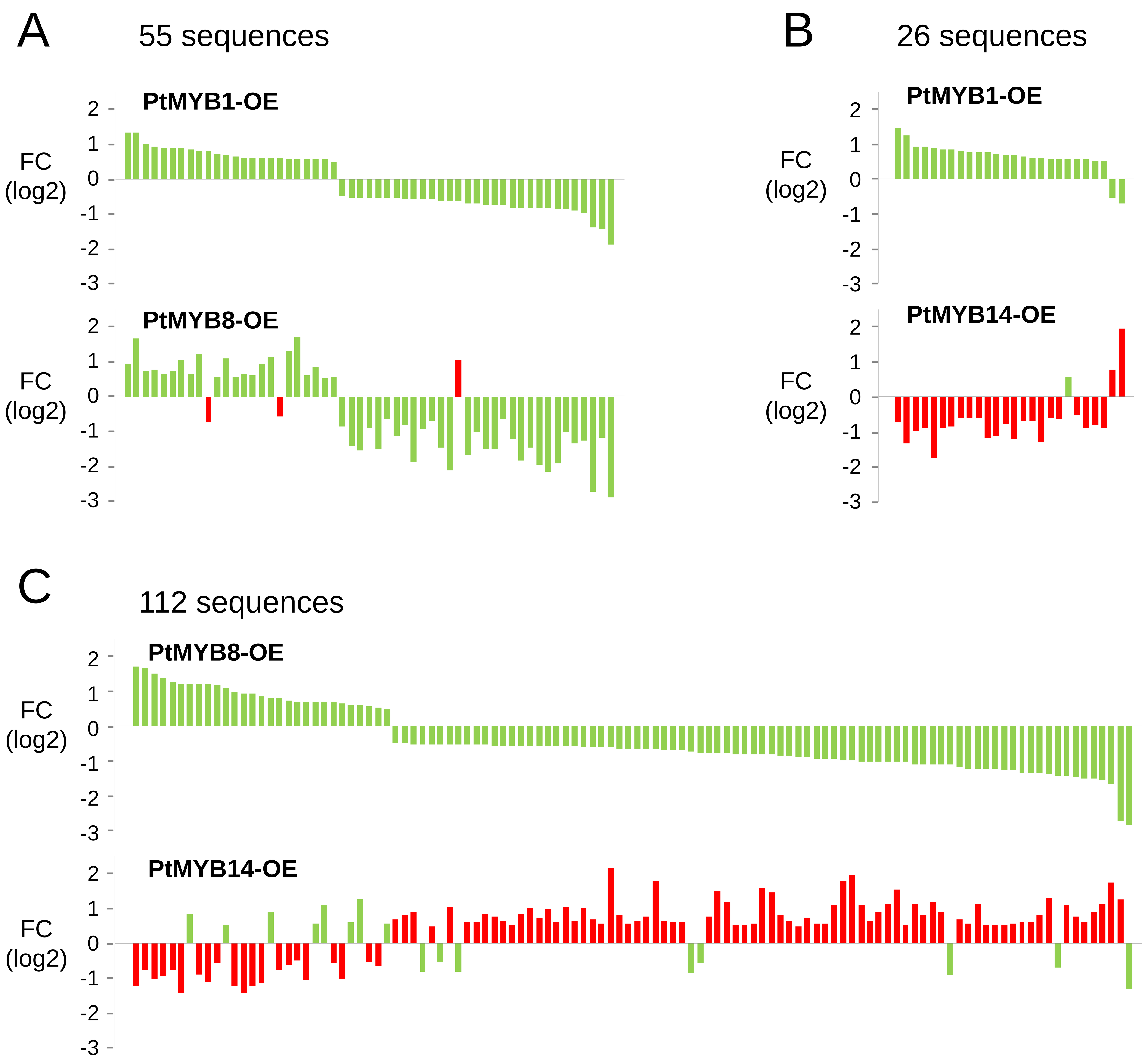

Fig. S1 Comparative analysis of microarray data associated to *Pinus taeda* MYB over-expression in spruce. Comparative fold-change (FC) expression levels for common mis-regulated sequences between (A) PtMYB1 versus PtMYB8, (B) PtMYB1 versus PtMYB14, and (C) PtMYB8 versus PtMYB14 comparison. In each comparison, red bar indicate an opposite expression. Complete information on sequence is reported in Supplementary Tables S1 and S2 at *JXB* online.
